# Associations between Methylenetetrahydrofolate Reductase (MTHFR) Polymorphisms and Non-Alcoholic Fatty Liver Disease (NAFLD) Risk: A Meta-Analysis

**DOI:** 10.1371/journal.pone.0154337

**Published:** 2016-04-29

**Authors:** Man-Yi Sun, Li Zhang, Song-Li Shi, Jing-Na Lin

**Affiliations:** 1 Department of Gastroenterology, Tianjin Union Medicine Center & Tianjin People’s Hospital, Tianjin, P.R. China; 2 Department of Pathology, Tianjin Union Medicine Center & Tianjin People’s Hospital, Tianjin, China; 3 Department of Endocrinology, Tianjin Union Medicine Center & Tianjin People’s Hospital, Tianjin, P.R. China; University of Birmingham, UNITED KINGDOM

## Abstract

**Background:**

C677T and A1298C are the most common allelic variants of Methylenetetrahydrofolate Reductase (MTHFR) gene. The association between MTHFR polymorphisms and the occurrence of non-alcoholic fatty liver disease (NAFLD) remains controversial. This study was thus performed to examine whether MTHFR mutations are associated with the susceptibility to NAFLD.

**Methods:**

A first meta-analysis on the association between the MTHFR polymorphisms and NAFLD risks was carried out via Review Manager 5.0 and Stata/SE 12.0 software. The on-line databases, such as PubMed, EMBASE, CENTRAL, WOS, Scopus and EBSCOhost (updated to April 1^st^, 2016), were searched for eligible case-control studies. The odd radio (OR), 95% confidence interval (CI) and *P* value were calculated through Mantel-Haenszel statistics under random- or fixed-effect model.

**Results:**

Eight articles (785 cases and 1188 controls) contributed data to the current meta-analysis. For C677T, increased NAFLD risks were observed in case group under homozygote model (T/T vs C/C, OR = 1.49, 95% CI = 1.03~2.15, *P* = 0.04) and recessive model (T/T vs C/C+C/T, OR = 1.42, 95% CI = 1.07~1.88, *P* = 0.02), but not the other genetics models, compared with control group. For A1298C, significantly increased NAFLD risks were detected in allele model (C vs A, OR = 1.53, 95% CI = 1.13~2.07, *P* = 0.006), homozygote model (C/C vs A/A, OR = 2.81, 95% CI = 1.63~4.85, *P* = 0.0002), dominant model (A/C+C/C vs A/A, OR = 1.60, 95% CI = 1.06~2.41, *P* = 0.03) and recessive model (C/C vs A/A+A/C, OR = 2.08, 95% CI = 1.45~3.00, *P*<0.0001), but not heterozygote model.

**Conclusion:**

T/T genotype of MTHFR C677T polymorphism and C/C genotype of MTHFR A1298C are more likely to be associated with the susceptibility to NAFLD.

## Introduction

Human Methylenetetrahydrofolate Reductase (MTHFR) gene is located at chromosome 1p36.3 and contains 11 exons [1, 2]. As a kind of folate-metabolizing enzyme, MTHFR protein is essential for the methylation of homocysteine (Hcy) to methionine, through catalyzing the irreversible reduction of 5,10-methylenetetrahydrofolate to 5-methyltetrahydrofolate [3–6]. The abnormity of MTHFR structure or function can take part in the occurrence of Hyperhomocysteinemia [5–7]. Two polymorphic variants, including C677T (rs1801133) and A1298C (rs1801131), have been identified in MTHFR gene [8–11].

Non-alcoholic fatty liver disease (NAFLD), the most common chronic liver disease, is the hepatic manifestation of the metabolic syndrome without a history of excess alcohol consumption [12–14]. The hepatic pathology of NAFLD mainly consists of simple fatty liver, non-alcoholic steatohepatitis (NASH), fibrosis and cirrhosis [15–18]. And NASH was characterized by hepatocellular injury and inflammation [15–18]. The polymorphisms of several genes, such as Patatin-like phospholipase domain-containing 3 (PNPLA3), leptin receptor (LEPR) and MTHFR, were reported to be involved in the genetic susceptibility to NAFLD [19–21]. For MTHFR gene, conflicting results regarding its potential correlation with NAFLD were reported [22–30]. Here, we focus on the polymorphisms of human MTHFR and assessed its genetic association with NAFLD risks via a meta-analysis, a very powerful tool for integrating and analyzing the conflicting data from different studies [31].

To our knowledge, no meta-analysis on the association of MTHFR genetic variants and overall NAFLD risks has been reported. Hence, we first carried out a meta-analysis to investigate the relationship between MTHFR polymorphisms (C677T and A1298C) and susceptibility to NAFLD. Our finding showed that both C677T and A1298C polymorphisms of MTHFR gene might positively correlate to the risks of NAFLD.

## Methods

### Searching strategy

A computerized literature search was performed from the electronic databases, including PubMed, Excerpta Medica Database (EMBASE), Cochrane Central Register of Controlled Trials (CENTRAL), Web of Science (WOS), China National Knowledge Infrastructure (CNKI)/WANFANG, Scopus and EBSCOhost in April 1^st^, 2016. There was no language or region restriction. The combinations of following keywords were used: “Methylenetetrahydrofolate Reductase (NADPH)” or “Methylene-THF Reductase (NADPH)” or “MTHFR” or “Methylenetetra hydrofolate Reductase”; “Non-alcoholic Fatty Liver Disease” or “NAFLD” or “Fatty Liver, Nonalcoholic” or “Livers, Nonalcoholic Fatty” or “Nonalcoholic Fatty Livers” or “Nonalcoholic Steatohepatitis” or “Nonalcoholic Steatohepatitides” or “Steatohepatitides, Nonalcoholic” or “Steatohepatitis, Nonalcoholic”; “Polymorphism, Genetic” or “Genetic Polymorphisms” or “Genetic Polymorphism” or “Polymorphism (Genetics)” or “Polymorphisms, Genetic”. The full details of databases searching terms were also provided (S1 Text).

### Inclusion and exclusion criteria

The eligible case-control studies were identified according to the following inclusion and exclusion criteria. Inclusion criteria: 1) The data on the association between MTHFR polymorphisms and susceptibility to NAFLD was provided; 2) The individual genotype frequencies for MTHFR polymorphisms could be extracted. Exclusion criteria: 1) duplicated studies; 2) reviews or books; 3) non-clinical data; 4) other genes; 5) non-NAFLD diseases; 6) case, trial, or non-polymorphism; 7) meeting/conference abstracts; 8) unavailable data.

### Data extraction strategy

Data was extracted from qualified articles independently by the authors (MYS LZ SLS JNL) using the same reporting form. The controversial evaluations were resolved through discussion. If the data was unavailable, an attempt was made to contact corresponding author to request missing data via E-mail. The following information was extracted: mutation site, first author, year of publication, country, ethnicity, sample sizes in case and control group, source of control, genotyping method, gender and age in case group, disease group, allele and genotype frequencies in each group, The Χ^2^ and *P* value of Hardy-Weinberg Equilibrium (HWE) test in control group. HWE value was calculated by chi-squared test and *P* value less than 0.05 was considered a departure from HWE.

### Statistical analysis

The *P* value, odd radio (OR) and corresponding 95% confidence interval (CI) were calculated by Mantel-Haenszel statistics under the allele, homozygote, heterozygote, dominant or recessive models. *P* value <0.05 was considered statistically significant association between C677T and A1298C polymorphisms of MTHFR and NAFLD risks. Χ^2^-based Q statistic and I^2^ test were applied to analyze the overall heterogeneities. When I^2^ values < 25% or *P* value of heterogeneity >0.10, a fixed-effect model was selected for Mantel-Haenszel statistics. Otherwise, a random-effect model was used [32–35]. When significant heterogeneity existed, sensitivity analysis was also performed to analyze the study that influenced homogeneity of the included studies. The potential publication bias was evaluated by Begg’s funnel plot with pseudo 95% confidence limits [36]. Statistical analyses were conducted by Review Manager Version 5.0 (The Nordic Cochrane Centre, The Cochrane Collaboration, Denmark) and Stata/SE 12.0 (StataCorp, College Station, USA) software.

## Results

### Study inclusion and characteristics

**Fig 1:**
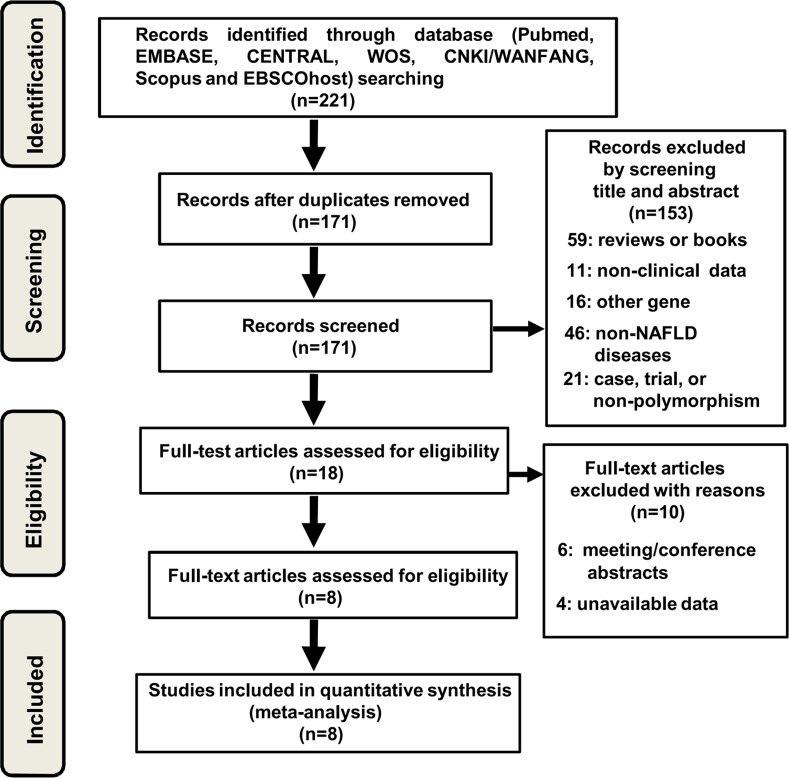
Flow chart of eligible studies selection during meta-analysis.

We searched the on-line electronic databases, including PubMed, EMBASE, CENTRAL, WOS, CNKI/WANFANG, Scopus and EBSCOhost (updated to April 1^st^, 2016), to obtain the eligible case-control studies. Flow chart of studies selection in meta-analysis was shown in Fig 1.

Possibly relevant articles of 221 were obtained from the electronic databases, including PubMed (n = 10), EMBASE (n = 29), CENTRAL (n = 0), WOS (n = 24), CNKI/WANFANG (n = 3), Scopus (n = 144) and EBSCOhost (n = 11). After 50 duplicated articles were removed, the 153 articles were excluded by screening the title and abstract: 59 articles are reviews or books; 11 articles do not provide the clinical data; 16 articles are related to the other genes; 46 articles focus on non-NAFLD diseases; 21 articles are case, trial or fail to contain the data of gene polymorphism. 18 potentially articles were then assessed for eligibility. The data was extracted from all these full-text articles. As shown in S2 Text, 6 articles were meeting/conference abstracts and 4 articles were lack of usable data. We failed to obtain missing data. Finally, 8 articles (785 cases and 1188 controls) fulfilled the inclusion criteria and were included in the present meta-analysis [23–30]. The data was extracted independently by the authors (MYS LZ SLS JNL). The characteristics of included articles were summarized and showed in Table 1. All the case-control studies were population-based. This meta-analysis was carried out according to the recommendations of the “Preferred Reporting Items for Systematic Reviews and Meta-Analyses” (PRISMA) statement (S1 Table) and “Meta-analysis on Genetic Association Studies” statement (S2 Table) [37].

**Table 1:** Characteristics of eligible studies in meta-analysis.

first author	year	country	ethnicity	sample sizes		source of control	genotying method	case	
				case	control			gender (male %)	age (year)
Chen et al.	2014	China	Asian	212	175	PB	PCR-gene CHIP	60.8	40~54
de Carvalho et al.	2013	Brazil	Caucasian	35	45	PB	PCR-RFLP/PCR-ASA	25.7	mean 49
Franco et al.	2013	Brazil	Caucasian	134	134	PB	PCR-RFLP	42.5	32~56
Hu et al.	2009	China	Asian	63	52	PB	PCR-RFLP	NA	NA
Kasapoglu et al.	2015	Turkey	Caucasian	150	136	PB	PCR-SSCP	30.0	32~63
Orlovskiy et al.	2015	Ukraine	Caucasian	100	40	PB	PCR-fluorescence hybridization	NA	NA
Sazci et al.	2008	Turkey	Caucasian	57	324	PB	PCR-RFLP	54.4	18~66
Serin et al.	2007	Turkey	Caucasian	34	282	PB	PCR-RFLP	55.9	33~51

PB: population-based; NA: not available; PCR-RFLP: polymerase chain reaction–restriction fragment length polymorphism; PCR-ASA: polymerase chain reaction–amplicon sequence analysis; PCR-SSCP: Polymerase chain reaction-single strand conformation polymorphism.

### Meta-analysis on the association between NAFLD risks and C677T polymorphism of MTHFR

**Fig 2:**
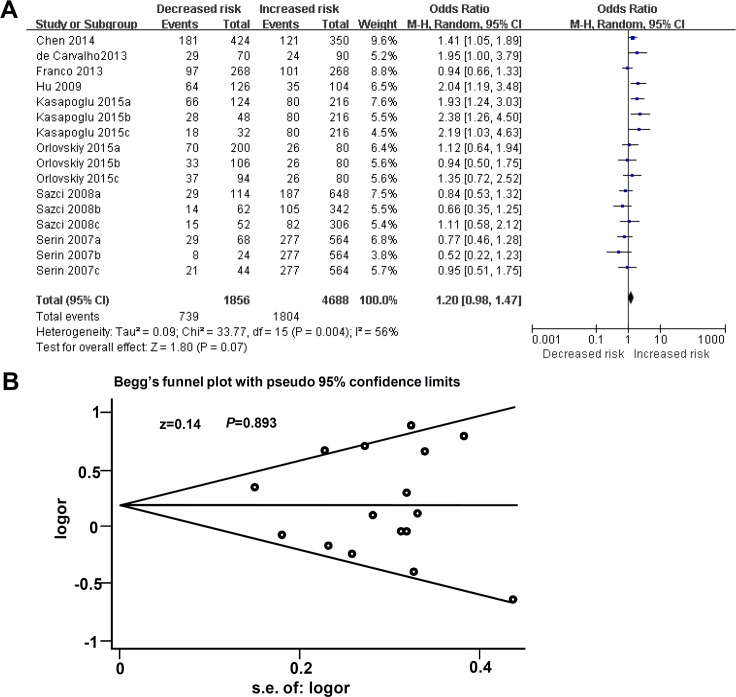
Meta analysis for the association between C677T allele frequency of MTHFR and the risks of NAFLD. (A) Forest plot under T vs C model; (B) Begg’s funnel plot of publication biases under T vs C model.

Next, the genetic association between MTHFR C677T polymorphism and susceptibility to NAFLD was measured. As shown in Fig 2A, the result (I^2^ = 56% and *P* = 0.004) revealed that high heterogeneity among studies was detected for C677T polymorphism. Random-effect model was thus applied for meta-analysis. The data on the association between C677T allele frequency of MTHFR and susceptibility to NAFLD was obtained (T vs C, OR = 1.20, 95% CI = 0.98~1.47, *P* = 0.07). In addition, the potential publication bias was evaluated by Begg’s funnel plot with pseudo 95% confidence limits. The result of Fig 2B suggested that basically symmetric plot (z = 0.14, *P* = 0.893) excludes the presence of large publication bias.

**Fig 3:**
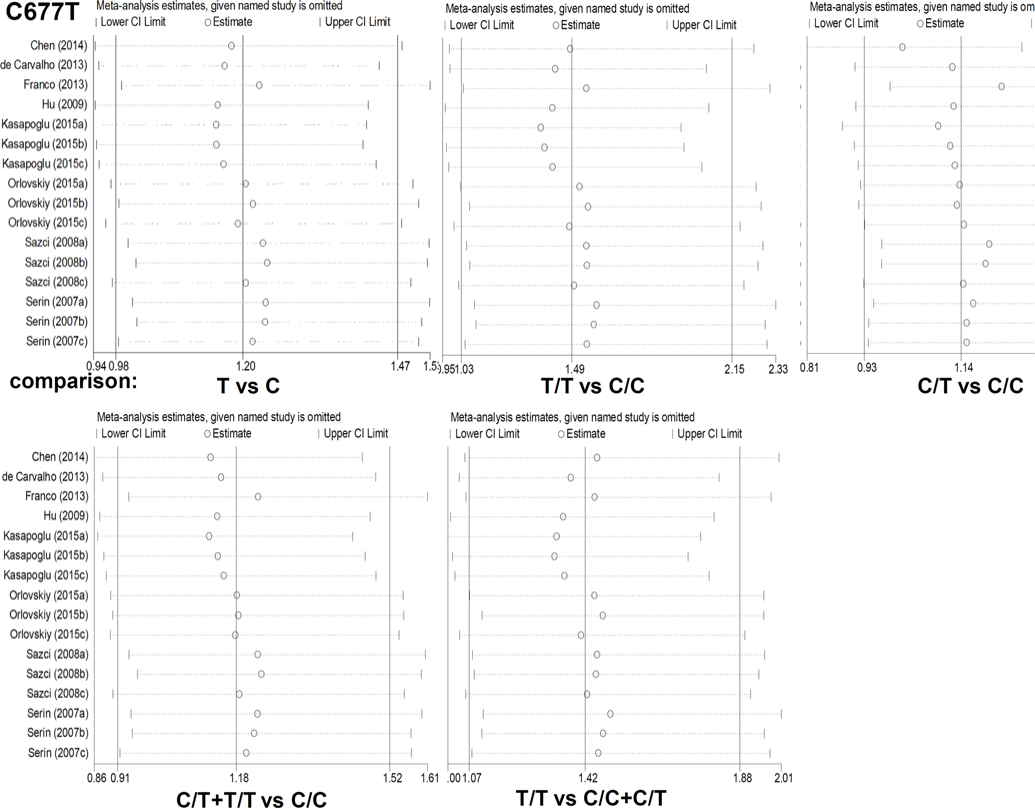
The sensitivity meta-analyses for the association between C677T polymorphism of MTHFR and the risks of NAFLD.

The contrast of the homozygote model (T/T vs C/C), heterozygote model (C/T vs C/C), dominant model (C/T+T/T vs C/C) and recessive model (T/T vs C/C+C/T) was then detected respectively, through the meta-analysis, in that the data on genotype frequencies of MTHFR C677T polymorphism was available. Genotype distribution and characteristics of MTHFR C677T polymorphism in different case-control studies were shown in Table 2. The T/T vs C/C (I^2^ = 47% and *P* = 0.02), C/T+T/T vs C/C (I^2^ = 45% and *P* = 0.03) and T/T vs C/C+C/T (I^2^ = 26% and *P* = 0.16) data indicated the existence of the moderate degree of heterogeneity across studies (Table 3). A random-effect model was thus used. However, fixed-effect model was used for the C/T vs C/C model (I^2^ = 21% and *P* = 0.21). Pooled analysis for the association between C677T genotype frequencies and the risks of NAFLD was shown in Table 3. Briefly, compared with control group, an increased NAFLD risk was observed in case group under homozygote model (T/T vs C/C, OR = 1.49, 95% CI = 1.03~2.15, *P* = 0.04) and recessive model (T/T vs C/C+C/T, OR = 1.42, 95% CI = 1.07~1.88, *P* = 0.02), but not the other genetics models (C/T vs C/C, OR = 1.14, 95% CI = 0.93~1.39, *P* = 0.21; C/T+T/T vs C/C, OR = 1.18, 95% CI = 0.91~1.52, *P* = 0.21). In addition, the results of HWE test (Table 2) in control group of two studies [24, 25] indicated that the genotype distributions deviated from HWE (Χ^2^ = 5.605, *P* = 0.018; Χ^2^ = 190.839, *P*<0.05). The subgroup analyses under all genetic models were also performed based on ethnicity or HWE (Table 4) via Stata/SE 12.0 software. A significantly increased NAFLD risk was observed in Asian population (T vs C, OR = 1.58, 95% CI = 1.13~2.20, *P* = 0.007; T/T vs C/C, OR = 1.97, 95% CI = 1.15~3.37, *P* = 0.014; C/T vs C/C, OR = 1.72, 95% CI = 1.16~2.55, *P* = 0.007; C/T+T/T vs C/C, OR = 1.81, 95% CI = 1.26~2.59, *P* = 0.001) and HWE *P*>0.05 subgroup (T vs C, OR = 1.31, 95% CI = 1.03~1.67, *P* = 0.030; T/T vs C/C, OR = 1.85, 95% CI = 1.15~2.97, *P* = 0.011; T/T vs C/C+C/T, OR = 1.72, 95% CI = 1.21~2.46, *P* = 0.003). In order to evaluate the influence of each study on the overall OR under all genetic models, the sensitivity meta-analyses, in which one study is omitted at a time, were also performed. As shown in Fig 3, the results indicated that the corresponding pooled OR value did not differ significantly from that of the overall meta-analysis. Furthermore, no significant publication bias was observed in all above genetic models via Begg’s funnel plot and Egger’s test (Data not shown), suggesting these results are reliable. These data indicated that the T/T genotype of MTHFR C677T polymorphism seems to be associated with genetic susceptibility to NAFLD, especially in Asian population.

**Table 2:** Genotype distribution of MTHFR C677T and A1298C polymorphisms.

site	first author	year	group	case				control				HWE	
				A/A	A/B	B/B	total	A/A	A/B	B/B	total	Χ^2^	*P*
**C677T**	de Carvalho	2013	-	12	17	6	35	23	20	2	45	0.837	0.360
	Franco	2013	-	57	57	20	134	50	67	17	134	0.559	0.455
	Chen	2014	-	71	101	40	212	82	65	28	175	5.605	**0.018**
	Kasapoglu	2015	C677T-stage 1	12	34	16	62	40	56	12	108	1.349	0.245
			C677T-stage 2	4	12	8	24	40	56	12	108		
			C677T-stage 3	3	8	5	16	40	56	12	108		
	Hu	2009	-	21	20	22	63	26	17	9	52	3.732	0.053
	Orlovskiy	2015	C677T-NAFLD-all	46	38	16	100	20	14	6	40	1.637	0.201
			C677T-NAFLD-only	25	23	5	53	20	14	6	40		
			C677T-NAFLD-T2D	21	15	11	47	20	14	6	40		
	Sazci	2008	NASH-overall	32	21	4	57	161	139	24	324	0.651	0.420
			NASH-men	19	10	2	31	81	75	15	171	0.162	0.688
			NASH-women	13	11	2	26	80	64	9	153	0.671	0.413
	Serin	2007	NAFLD	19	1	14	34	131	25	126	282	190.839	**<0.05**
			NAFL	8	0	4	12	131	25	126	282		
			NASH	11	1	10	22	131	25	126	282		
**A1298C**	de Carvalho	2013	-	20	15	0	35	26	17	2	45	0.141	0.708
	Franco	2013	-	74	53	7	134	65	63	6	134	3.711	0.054
	Kasapoglu	2015	A1298C-stage 1	12	18	8	38	40	24	4	68	0.025	0.874
			A1298C-stage 2	4	8	6	18	40	24	4	68		
			A1298C-stage 3	3	5	3	11	40	24	4	68		
	Orlovskiy	2015	A1298C-NAFLD-all	53	34	13	100	20	17	3	40	0.056	0.813
			A1298C-NAFLD-only	32	17	4	53	20	17	3	40		
			A1298C-NAFLD-T2D	22	16	9	47	20	17	3	40		
	Sazci	2008	NASH-all	13	34	10	57	137	154	33	324	1.159	0.282
			NASH-men	7	20	4	31	73	79	19	171	0.119	0.731
			NASH-women	6	14	6	26	64	75	14	153	1.458	0.227

NA: not available; HWE: Hardy-Weinberg Equilibrium.

**Table 3:** Pooled analysis for the association between MTHFR C677T genotype frequencies and the risks of NAFLD.

Comparison	study	case		control		Test of association		Heterogeneity		Model
		Events	Total	Events	Total	OR (95% CI)	*P* value	I^2^ (%)	*P* value	
**T/T vs C/C (homozygote)**		**T/T**		**T/T**		**1.49 [1.03, 2.15]**	**0.04**	**47**	**0.02**	**R**
	de Carvalho,2013	6	18	2	25	5.75 [1.00, 32.95]				
	Franco,2013	20	77	17	67	1.03 [0.49, 2.18]				
	Chen,2014	40	111	28	110	1.65 [0.93, 2.94]				
	Kasapoglu,2015	16	28	12	52	4.44 [1.65, 11.94]				
		8	12	12	52	6.67 [1.71, 26.04]				
		5	8	12	52	5.56 [1.16, 26.70]				
	Hu,2009	22	43	9	35	3.03 [1.15, 7.95]				
	Orlovskiy,2015	16	62	6	26	1.16 [0.40, 3.40]				
		5	30	6	26	0.67 [0.18, 2.51]				
		11	32	6	26	1.75 [0.54, 5.62]				
	Sazci,2008	4	36	24	185	0.84 [0.27, 2.58]				
		2	21	15	96	0.57 [0.12, 2.70]				
		2	15	9	89	1.37 [0.27, 7.05]				
	Serin,2007	14	33	126	257	0.77 [0.37, 1.59]				
		4	12	126	257	0.52 [0.15, 1.77]				
		10	21	126	257	0.95 [0.39, 2.30]				
**C/T vs C/C (heterozygote)**		**C/T**		**C/T**		**1.14 [0.93, 1.39]**	**0.21**	**21**	**0.21**	**F**
	de Carvalho,2013	17	29	20	43	1.63 [0.63, 4.22]				
	Franco,2013	57	114	67	117	0.75 [0.44, 1.25]				
	Chen,2014	101	172	65	147	1.79 [1.15, 2.80]				
	Kasapoglu,2015	34	46	56	96	2.02 [0.93, 4.38]				
		12	16	56	96	2.14 [0.64, 7.13]			
		8	11	56	96	1.90 [0.48, 7.63]					
	Hu,2009	20	41	17	43	1.46 [0.61, 3.46]					
	Orlovskiy,2015	38	84	14	34	1.18 [0.53, 2.64]					
		23	48	14	34	1.31 [0.54, 3.19]					
		15	36	14	34	1.02 [0.39, 2.64]					
	Sazci,2008	21	53	139	300	0.76 [0.42, 1.38]					
		10	29	75	156	0.57 [0.25, 1.30]					
		11	24	64	144	1.06 [0.44, 2.52]					
	Serin,2007	1	20	25	156	0.28 [0.04, 2.15]					
		0	8	25	156	0.30 [0.02, 5.42]					
		1	12	25	156	0.48 [0.06, 3.86]					
**C/T+T/T vs C/C (dominant)**		**C/T+T/T**		**C/T+T/T**		**1.18 [0.91, 1.52]**	**0.21**	**45**	**0.03**	**R**	
	de Carvalho,2013	23	35	22	45	2.00 [0.81, 4.98]					
	Franco,2013	77	134	84	134	0.80 [0.49, 1.31]					
	Chen,2014	141	212	93	175	1.75 [1.16, 2.64]					
	Kasapoglu,2015	50	62	68	108	2.45 [1.17, 5.14]					
		20	24	68	108	2.94 [0.94, 9.22]					
		13	16	68	108	2.55 [0.68, 9.49]					
	Hu,2009	42	63	26	52	2.00 [0.94, 4.25]					
	Orlovskiy,2015	54	100	20	40	1.17 [0.56, 2.45]					
		28	53	20	40	1.12 [0.49, 2.55]					
		26	47	20	40	1.24 [0.53, 2.88]					
	Sazci,2008	25	57	163	324	0.77 [0.44, 1.36]					
		12	31	90	171	0.57 [0.26, 1.24]					
		13	26	73	153	1.10 [0.48, 2.52]					
	Serin,2007	15	34	151	282	0.68 [0.33, 1.40]					
		4	12	151	282	0.43 [0.13, 1.47]					
		11	22	151	282	0.87 [0.36, 2.07]					
**T/T vs C/C+C/T (recessive)**		**T/T**		**T/T**		**1.42 [1.07, 1.88]**	**0.02**	**26**	**0.16**	**R**	
	de Carvalho,2013	6	35	2	45	4.45 [0.84, 23.59]					
	Franco,2013	20	134	17	134	1.21 [0.60, 2.42]					
	Chen,2014	40	212	28	175	1.22 [0.72, 2.08]					
	Kasapoglu,2015	16	62	12	108	2.78 [1.22, 6.36]					
		8	24	12	108	4.00 [1.41, 11.31]					
		5	16	12	108	3.64 [1.08, 12.26]					
	Hu,2009	22	63	9	52	2.56 [1.06, 6.22]					
	Orlovskiy,2015	16	100	6	40	1.08 [0.39, 2.99]					
		5	53	6	40	0.59 [0.17, 2.09]					
		11	47	6	40	1.73 [0.58, 5.20]					
	Sazci,2008	4	57	24	324	0.94 [0.31, 2.83]					
		2	31	15	171	0.72 [0.16, 3.30]					
		2	26	9	153	1.33 [0.27, 6.55]					
	Serin,2007	14	34	126	282	0.87 [0.42, 1.78]					
		4	12	126	282	0.62 [0.18, 2.10]					
		10	22	126	282	1.03 [0.43, 2.47]					

R: Random-effect; F:Fixed-effect.

**Table 4:** Subgroup analysis for the association between MTHFR C677T genotype frequencies and the risks of NAFLD.

Comparison	subgroup	Number of studies	Sample size		OR (95% CI)	*P* value
			case	control		
**T vs C (allele)**	**Ethnicity**					
	Asian	2	275	227	1.58 [1.13, 2.20]	**0.007**
	Caucasian	14	653	2117	1.14 [0.91, 1.42]	0.269
	**HWE**					
	*P*<0.05	4	280	1021	0.94 [0.62, 1.44]	0.778
	*P*>0.05	12	648	1323	1.31 [1.03, 1.67]	**0.030**
**T/T vs C/C (homozygote)**	**Ethnicity**					
	Asian	2	275	227	1.97 [1.15, 3.37]	**0.014**
	Caucasian	14	653	2117	1.39 [0.90, 2.13]	0.134
	**HWE**					
	*P*<0.05	4	280	1021	1.01 [0.63, 1.64]	0.954
	*P*>0.05	12	648	1323	1.85 [1.15, 2.97]	**0.011**
**C/T vs C/C (heterozygote)**	**Ethnicity**					
	Asian	2	275	227	1.72 [1.16, 2.55]	**0.007**
	Caucasian	14	653	2117	0.99 [0.78, 1.24]	0.914
	**HWE**					
	*P*<0.05	4	280	1021	1.39 [0.93, 2.07]	0.108
	*P*>0.05	12	648	1323	1.07 [0.85, 1.34]	0.581
**C/T+T/T vs C/C (dominant)**	**Ethnicity**					
	Asian	2	275	227	1.81 [1.26, 2.59]	**0.001**
	Caucasian	14	653	2117	1.07 [0.82, 1.39]	0.635
	**HWE**					0.211
	*P*<0.05	4	280	1021	0.93 [0.49, 1.75]	0.814
	*P*>0.05	12	648	1323	1.25 [0.93, 1.67]	0.138
**T/T vs C/C+C/T (recessive)**	**Ethnicity**					
	Asian	2	275	227	1.62 [0.80, 3.28]	0.181
	Caucasian	14	653	2117	1.38 [0.99, 1.92]	0.060
	**HWE**					
	*P*<0.05	4	280	1021	1.02 [0.71, 1.47]	0.912
	*P*>0.05	12	648	1323	1.72 [1.21, 2.46]	**0.003**

HWE: Hardy-Weinberg Equilibrium.

### Meta-analysis on the association between NAFLD risks and A1298C polymorphism of MTHFR

**Fig 4:**
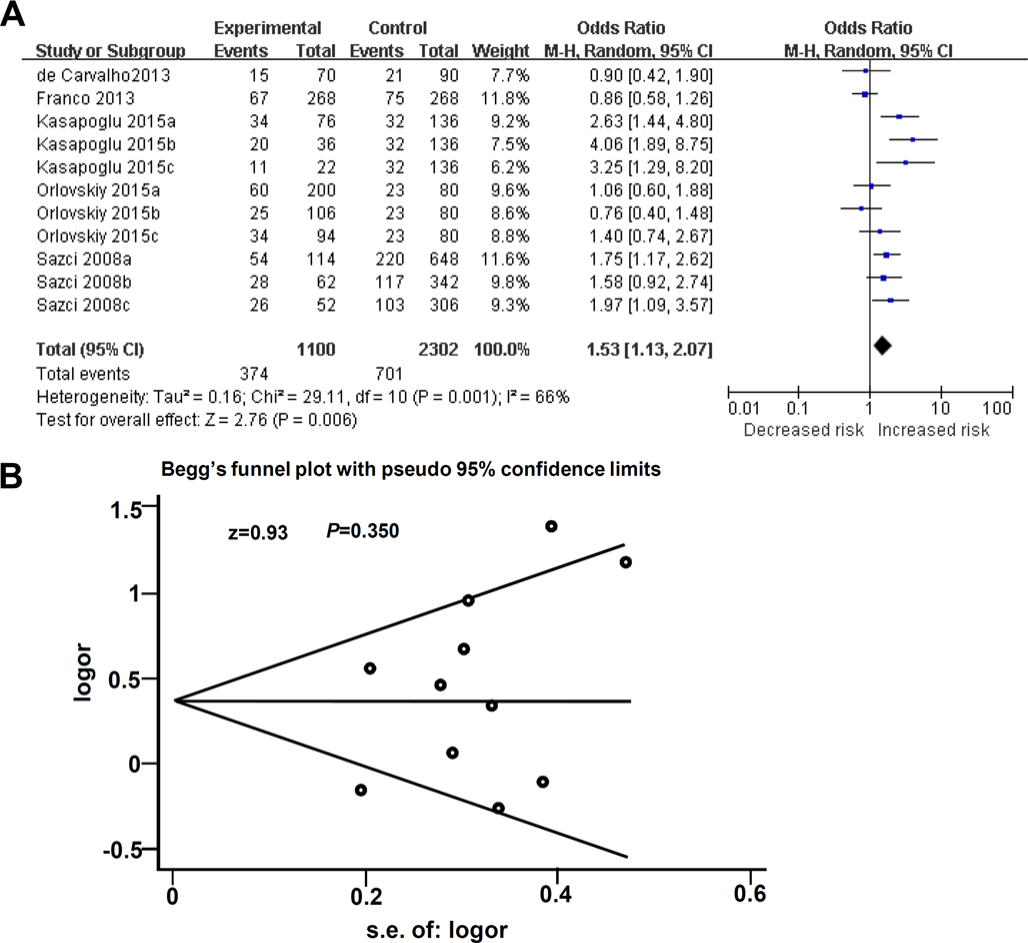
Meta analysis for the association between A1298C allele frequency of MTHFR and the risks of NAFLD. (A) Forest plot under C vs A model; (B) Begg’s funnel plot of publication biases under C vs A model.

Besides C677T, meta-analysis on the association between MTHFR A1298C polymorphism and NAFLD risks was also performed. Table 2 showed the genotype distribution and characteristics of MTHFR A1298C polymorphism. All the control groups of these studies were in line with HWE (All *P*>0.05). In addition, all the case-control studies were performed in Caucasian population. We then first performed the meta-analysis between the allele frequency of MTHFR A1298C and the susceptibility to NAFLD under C vs A model. As shown in Fig 4A, random-effect model was used, due to the existence of high between-studies heterogeneity (I^2^ = 66% and *P* = 0.001) for meta-analysis. The data (OR = 1.53, 95% CI = 1.13~2.07, *P* = 0.006) was obtained in C vs A comparison of MTHFR A1298C. The basically symmetric plot (z = 0.93, *P* = 0.350) did not provide the statistical evidence for publication bias (Fig 4B).

**Fig 5:**
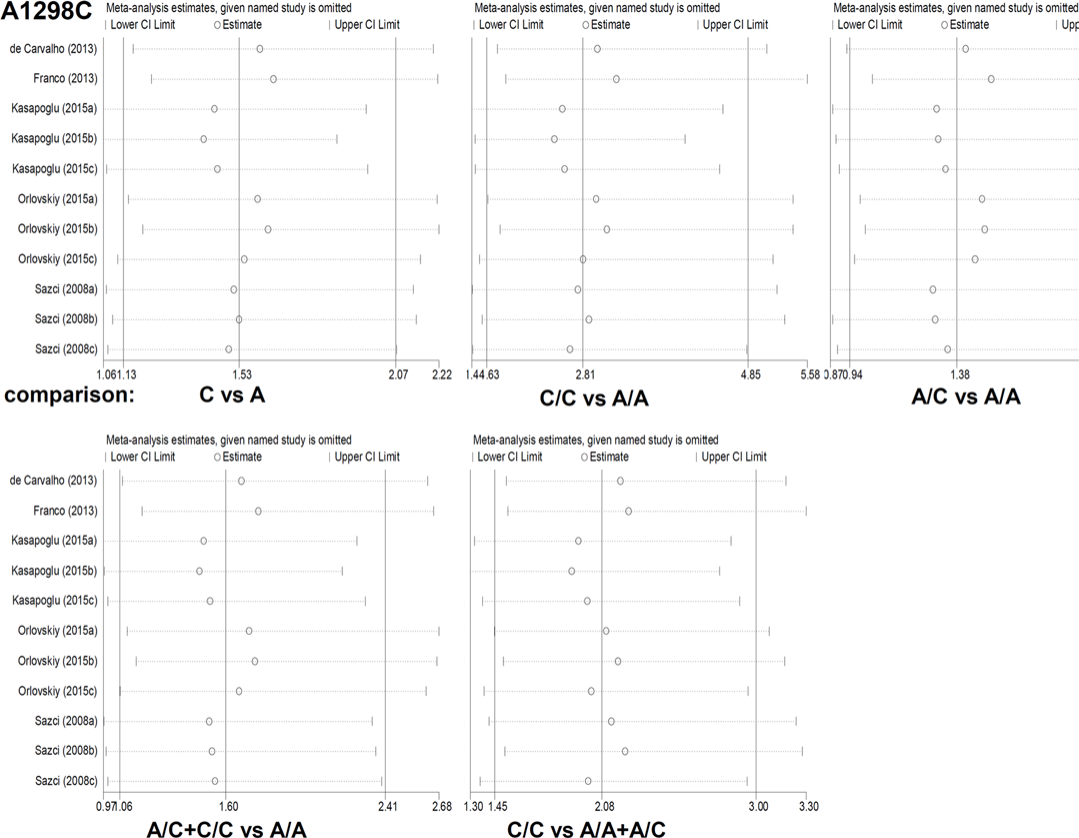
The sensitivity meta-analyses for the association between A1298C polymorphism of MTHFR and the risks of NAFLD.

Moreover, we also performed the pooled analysis for the associations between MTHFR genotype frequencies of A1298C and the susceptibility to NAFLD (Table 5). The data of C/C vs A/A model (I^2^ = 39% and *P* = 0.09), A/C vs A/A model (I^2^ = 53% and *P* = 0.02), A/C+C/C vs A/A model (I^2^ = 63% and *P* = 0.003) was obtained and random-effect model was used. For the C/C vs A/A+A/C model, fixed-effect model was used (I^2^ = 14% and *P* = 0.31). A significantly increased NAFLD risks was observed in homozygote model (C/C vs A/A, OR = 2.81, 95% CI = 1.63~4.85, *P* = 0.0002), dominant model (A/C+C/C vs A/A, OR = 1.60, 95% CI = 1.06~2.41, P = 0.03) and recessive models (C/C vs A/A+A/C, OR = 2.08, 95% CI = 1.45~3.00, *P*<0.0001), but not heterozygote model (A/C vs A/A, OR = 1.38, 95% CI = 0.94~2.03, *P* = 0.10). Moreover, similar results were obtained in the sensitivity meta-analyses under all genetic models (Fig 5). These data suggested that C/C genotype of MTHFR A1298C polymorphism is more likely to be strongly associated with the susceptibility to NAFLD in Caucasian population.

**Table 5:** Pooled analysis for the association between MTHFR A1298C genotype frequencies and the risks of NAFLD.

Comparison	study	case		control		Test of association		Heterogeneity		Model
		Events	Total	Events	Total	OR (95% CI)	*P* value	I^2^ (%)	*P* value	
**C/C vs A/A (homozygote)**		**C/C**		**C/C**		**2.81 [1.63, 4.85]**	**0.0002**	**39**	**0.09**	**R**
	de Carvalho,2013	0	20	2	28	0.26 [0.01, 5.69]				
	Franco,2013	7	81	6	71	1.02 [0.33, 3.20]				
	Kasapoglu,2015	8	20	4	44	6.67 [1.71, 26.04]				
		6	10	4	44	15.00 [2.94, 76.56]				
		3	6	4	44	10.00 [1.49, 66.99]				
	Orlovskiy,2015	13	66	3	23	1.64 [0.42, 6.35]				
		4	36	3	23	0.83 [0.17, 4.12]				
		9	31	3	23	2.73 [0.65, 11.51]				
	Sazci,2008	10	23	33	170	3.19 [1.29, 7.92]				
		4	11	19	92	2.20 [0.58, 8.29]				
		6	12	14	78	4.57 [1.28, 16.29]				
**A/C vs A/A (heterozygote)**		**A/C**		**A/C**		**1.38 [0.94, 2.03]**	**0.10**	**53**	**0.02**	**R**
	de Carvalho,2013	15	35	17	43	1.15 [0.46, 2.84]				
	Franco,2013	53	127	63	128	0.74 [0.45, 1.21]				
	Kasapoglu,2015	18	30	24	64	2.50 [1.03, 6.08]				
		8	12	24	64	3.33 [0.91, 12.26]				
		5	8	24	64	2.78 [0.61, 12.68]				
	Orlovskiy,2015	34	87	17	37	0.75 [0.35, 1.64]				
		17	49	17	37	0.63 [0.26, 1.50]				
		16	38	17	37	0.86 [0.34, 2.13]				
	Sazci,2008	34	47	154	291	2.33 [1.18, 4.59]				
		20	27	79	152	2.64 [1.05, 6.61]				
		14	20	75	139	1.99 [0.72, 5.48]				
**A/C+C/C vs A/A (dominant)**		**A/C+C/C**		**A/C+C/C**		**1.60 [1.06, 2.41]**	**0.03**	**63**	**0.003**	**R**
	de Carvalho,2013	15	35	19	45	1.03 [0.42, 2.51]				
	Franco,2013	60	134	69	134	0.76 [0.47, 1.23]				
	Kasapoglu,2015	26	38	28	68	3.10 [1.34, 7.15]				
		14	18	28	68	5.00 [1.49, 16.79]				
		8	11	28	68	3.81 [0.93, 15.64]				
	Orlovskiy,2015	47	100	20	40	0.89 [0.43, 1.85]				
		21	53	20	40	0.66 [0.29, 1.50]				
		25	47	20	40	1.14 [0.49, 2.64]				
	Sazci,2008	44	57	187	324	2.48 [1.29, 4.78]				
		24	31	98	171	2.55 [1.04, 6.25]				
		20	26	89	153	2.40 [0.91, 6.31]				
**C/C vs A/A+A/C (recessive)**		**C/C**		**C/C**		**2.08 [1.45, 3.00]**	**<0.0001**	**14**	**0.31**	**F**
	de Carvalho,2013	0	35	2	45	0.25 [0.01, 5.27]				
	Franco,2013	7	134	6	134	1.18 [0.38, 3.60]				
	Kasapoglu,2015	8	38	4	68	4.27 [1.19, 15.29]				
		6	18	4	68	8.00 [1.96, 32.68]				
		3	11	4	68	6.00 [1.13, 31.80]				
	Orlovskiy,2015	13	100	3	40	1.84 [0.50, 6.85]				
		4	53	3	40	1.01 [0.21, 4.78]				
		9	47	3	40	2.92 [0.73, 11.64]				
	Sazci,2008	10	57	33	324	1.88 [0.87, 4.06]				
		4	31	19	171	1.19 [0.37, 3.76]				
		6	26	14	153	2.98 [1.03, 8.64]				

R: Random-effect; F:Fixed-effect.

## Discussion

Several studies have reported the potential association between the most common allelic variants of MTHFR gene (C677T and A1298C) and susceptibility to many clinical diseases, such as gastric cancer, hepatocellular carcinoma, NAFLD, neural tube defects, acute lymphoblastic leukemia and renal/heart failure [11, 25, 26, 38–42]. For example, MTHFR C677T polymorphism is found to be linked to an increased risk of neural tube defects [40]; MTHFR gene mutations might be conductive to renal function in Italian population [42]. However, the effect of MTHFR polymorphisms in the presence of NAFLD remains inconclusive in different populations [22–30]. For instance, C677T and A1298C polymorphisms of MTHFR gene were significantly associated with NASH risks in Turkish population [26]. The association of MTHFR A1298C polymorphism with NAFLD severity was also observed in Italy population [22]. However, both MTHFR C677T and A1298C polymorphisms were not considered as the potential genetic risk factors for the development of NAFLD in Brazilian population [29]. The data of Serin et al also suggested that MTHFR C677T polymorphism is unlikely to be associated with the progression of non-alcoholic fatty liver to NASH in their Turkish cohort study [25]. Here, a meta-analysis was first conducted to further comprehensively evaluate the genetic association, based on the data from all available population-based case-control studies.

The positive correlation between NAFLD susceptibility and two MTHFR variants (C677T and A1298C) was observed in our statistical evidence. For C677T polymorphism, an increased NAFLD risk was observed under homozygote model (T/T vs C/C) and recessive model (T/T vs C/C+C/T), but not T vs C, C/T vs C/C and C/T+T/T vs C/C models, suggesting that T/T genotype of MTHFR C677T polymorphism might have the increased risks of NAFLD in general population. Moreover, we found that a significantly increased NAFLD risk was detected in Asian population under the comparison of T vs C, T/T vs C/C, C/T vs C/C; C/T+T/T vs C/C. Similarly, the meta-analysis of A1298C polymorphism based on 11 case-control studies in Caucasian population provided the evidence that a significantly increased NAFLD risk was observed under allele model (C vs A), homozygote model (C/C vs A/A), dominant model (A/C+ C/C vs A/C) and recessive model (C/C vs A/A+A/C), but not heterozygote model (A/C vs A/A), suggesting that C/C genotype of MTHFR A1298C polymorphism might be linked to the susceptibility to NAFLD in Caucasian population.

The C677T polymorphism means the substitution of C (cytosine) to T (thymine) at nucleotide position 677, which results in the transition from alanine to valine, while A1298C polymorphism refers to the transition of A (adenine) to C (cytosine) at position 1298, which leads to an amino acid substitution from glutamic acid to alanine [8–11]. Folate is closely associated with the synthesis, methylation and repair of DNA, and is essential for the production or maintenance of normal cell and the inhibition of tumor cells [43–45]. The mutations of MTHFR gene were reported to reduce the enzyme activity of MTHFR, concentration of folate, and thus take part in the up-regulation of serum Hcy levels [6, 46, 47]. Kasapoglu B, et al. reported that homozygote mutations of MTHFR C677T and A1298C are positively associated with the increased levels of serum Hcy in NAFLD individuals [28]. Here, individuals, who carry T/T genotype in C677T and C/C genotype in A1298C polymorphism, might have high risks of NAFLD. It is possible that the two harmful homozygous mutations of MTHFR gene confer susceptibility to NAFLD via the abnormity of MTHFR enzyme activity and folate-involved DNA metabolism. Intriguingly, homozygote C/C genotype of MTHFR A1298C seems to be significantly linked to a decreased risk of liver cancer in Asian population, whereas homozygote T/T genotype of MTHFR C677T shows a reversed effect [38, 48, 49]. More experiments are needed to investigate the molecular mechanism on the distinct roles of MTHFR polymorphisms in the occurrence of NAFLD and hepatic carcinoma.

There are some shortages or limitations in this meta-analysis, which should be pointed out. For example, no large sample size was included in the case/control groups of meta-analysis. It is still possible that other unpublished or undetected studies are present, although we selected the eligible studies independently according to the inclusion and exclusion criteria. The potential selection bias still may affect the reliability of our findings. Different degree of heterogeneity and departure from HWE was also detected in some comparisons or case-control studies. Furthermore, it was reported that C677C/C1298C compound genotype confers increased risks of NASH in Turkish women patients [26]. Unfortunately, we failed to carry out the meta-analysis to investigate the potential role of MTHFR susceptibility loci combination in the susceptibility to NAFLD, due to the limitation of relevant data.

Very complicated natural history of NAFLD was existed, and multiple genetic or environmental factors contribute to the occurrence and progression of the NAFLD [50–53]. NAFLD has become a public health concern for its close relation with the other metabolic syndrome, hyperhomocysteinemia, obesity, hypertension, type 2 diabetes mellitus, cardiovascular disease or hepatocellular carcinoma [50–54]. Accumulating evidence showed the relationship between the MTHFR polymorphism and the pathogenesis of NAFLD-associated diseases [38, 42, 55–57]. To perform more frequent screening of functional MTHFR gene variants and other potential clinical characteristics is useful to reduce the development of the above diseases. Larger and well-designed studies and further meta-analyses based on population feature, disease status, gender, geographical location, detailed information of diet or physical activity are required to study the role of MTHFR mutation in the risks of NAFLD and NAFLD-associated diseases.

### Conclusion

All in all, this is the first meta-analysis to provide evidence that C677T and A1298C mutations of MTHFR are significantly associated with an increased risk of NAFLD. The homozygous T/T genotype of MTHFR C677T and C/C genotype of MTHFR A1298C polymorphism seem to be more susceptible to NAFLD. More case-control studies are warranted to validate the conclusion.

## Supporting Information

S1 Table PRISMA 2009 checklist. (DOCX)

S2 Table meta-analysis on genetic association studies form. (DOCX)

S1 Text The full details of databases searching terms. (DOCX)

S2 Text Full-text articles excluded with reasons. (DOCX)

## References

[1] Goyette P, Sumner JS, Milos R, Duncan AM, Rosenblatt DS, Matthews RG, et al Human methylenetetrahydrofolate reductase: isolation of cDNA, mapping and mutation identification. Nat Genet. 1994;7(2):195–200. Epub 1994/06/01. 10.1038/ng0694-195 . 7920641

[2] Goyette P, Pai A, Milos R, Frosst P, Tran P, Chen Z, et al Gene structure of human and mouse methylenetetrahydrofolate reductase (MTHFR). Mamm Genome. 1998;9(8):652–656. Epub 1998/07/29. . 9680386 10.1007/s003359900838

[3] Santilli F, Davi G, Patrono C. Homocysteine, methylenetetrahydrofolate reductase, folate status and atherothrombosis: A mechanistic and clinical perspective. Vascul Pharmacol. 2015 Epub 2015/06/27. 10.1016/j.vph.2015.06.009 . 26111718

[4] Frosst P, Blom HJ, Milos R, Goyette P, Sheppard CA, Matthews RG, et al A candidate genetic risk factor for vascular disease: a common mutation in methylenetetrahydrofolate reductase. Nat Genet. 1995;10(1):111–113. Epub 1995/05/01. 10.1038/ng0595-111 . 7647779

[5] Rozen R. Genetic predisposition to hyperhomocysteinemia: deficiency of methylenetetrahydrofolate reductase (MTHFR). Thromb Haemost. 1997;78(1):523–526. Epub 1997/07/01. . 9198208

[6] Cardona H, Cardona-Maya W, Gomez JG, Castaneda S, Gomez JM, Bedoya G, et al Relationship between methylenetetrahydrofolate reductase polymorphism and homocysteine levels in women with recurrent pregnancy loss: a nutrigenetic perspective. Nutr Hosp. 2008;23(3):277–282. Epub 2008/06/19. . 18560705

[7] Moll S, Varga EA. Homocysteine and MTHFR Mutations. Circulation. 2015;132(1):e6–9. Epub 2015/07/08. 10.1161/circulationaha.114.013311 . 26149435

[8] Guenther BD, Sheppard CA, Tran P, Rozen R, Matthews RG, Ludwig ML. The structure and properties of methylenetetrahydrofolate reductase from Escherichia coli suggest how folate ameliorates human hyperhomocysteinemia. Nat Struct Biol. 1999;6(4):359–365. Epub 1999/04/14. 10.1038/7594 . 10201405

[9] van der Put NM, Gabreels F, Stevens EM, Smeitink JA, Trijbels FJ, Eskes TK, et al A second common mutation in the methylenetetrahydrofolate reductase gene: an additional risk factor for neural-tube defects? Am J Hum Genet. 1998;62(5):1044–1051. Epub 1998/05/23. 10.1086/301825 ; PubMed Central PMCID: PMCPmc1377082. 9545395 PMC1377082

[10] Liew SC, Gupta ED. Methylenetetrahydrofolate reductase (MTHFR) C677T polymorphism: epidemiology, metabolism and the associated diseases. Eur J Med Genet. 2015;58(1):1–10. Epub 2014/12/03. 10.1016/j.ejmg.2014.10.004 . 25449138

[11] Xia LZ, Liu Y, Xu XZ, Jiang PC, Ma G, Bu XF, et al Methylenetetrahydrofolate reductase C677T and A1298C polymorphisms and gastric cancer susceptibility. World J Gastroenterol. 2014;20(32):11429–11438. Epub 2014/08/30. 10.3748/wjg.v20.i32.11429 ; PubMed Central PMCID: PMCPmc4145786. 25170232 PMC4145786

[12] Basaranoglu M, Ormeci N. Nonalcoholic fatty liver disease: diagnosis, pathogenesis, and management. Turk J Gastroenterol. 2014;25(2):127–132. Epub 2014/07/09. 10.5152/tjg.2014.7675 . 25003670

[13] Chalasani N, Younossi Z, Lavine JE, Diehl AM, Brunt EM, Cusi K, et al The diagnosis and management of non-alcoholic fatty liver disease: practice Guideline by the American Association for the Study of Liver Diseases, American College of Gastroenterology, and the American Gastroenterological Association. Hepatology. 2012;55(6):2005–2023. Epub 2012/04/11. 10.1002/hep.25762 . 22488764

[14] Schreuder TC, Verwer BJ, van Nieuwkerk CM, Mulder CJ. Nonalcoholic fatty liver disease: an overview of current insights in pathogenesis, diagnosis and treatment. World J Gastroenterol. 2008;14(16):2474–2486. Epub 2008/04/30. ; PubMed Central PMCID: PMCPmc2708357. 18442193 10.3748/wjg.14.2474 PMC2708357

[15] Clark JM. The epidemiology of nonalcoholic fatty liver disease in adults. J Clin Gastroenterol. 2006;40 Suppl 1:S5–10. Epub 2006/03/17. 10.1097/01.mcg.0000168638.84840.ff . 16540768

[16] Angulo P. GI epidemiology: nonalcoholic fatty liver disease. Aliment Pharmacol Ther. 2007;25(8):883–889. Epub 2007/04/04. 10.1111/j.1365-2036.2007.03246.x . 17402991

[17] Dowman JK, Tomlinson JW, Newsome PN. Pathogenesis of non-alcoholic fatty liver disease. Qjm. 2010;103(2):71–83. Epub 2009/11/17. 10.1093/qjmed/hcp158 ; PubMed Central PMCID: PMCPmc2810391. 19914930 PMC2810391

[18] Petta S, Muratore C, Craxi A. Non-alcoholic fatty liver disease pathogenesis: the present and the future. Dig Liver Dis. 2009;41(9):615–625. Epub 2009/02/19. 10.1016/j.dld.2009.01.004 . 19223251

[19] Macaluso FS, Maida M, Petta S. Genetic background in nonalcoholic fatty liver disease: A comprehensive review. World J Gastroenterol. 2015;21(39):11088–11111. 10.3748/wjg.v21.i39.11088 26494964 PMC4607907

[20] Lim JW, Dillon J, Miller M. Proteomic and genomic studies of non-alcoholic fatty liver disease—clues in the pathogenesis. World J Gastroenterol. 2014;20(26):8325–8340. Epub 2014/07/16. 10.3748/wjg.v20.i26.8325 ; PubMed Central PMCID: PMCPmc4093687. 25024592 PMC4093687

[21] Speliotes EK, Yerges-Armstrong LM, Wu J, Hernaez R, Kim LJ, Palmer CD, et al Genome-wide association analysis identifies variants associated with nonalcoholic fatty liver disease that have distinct effects on metabolic traits. PLoS Genet. 2011;7(3):e1001324 10.1371/journal.pgen.1001324 21423719 PMC3053321

[22] Catalano D, Trovato GM, Ragusa A, Martines GF, Tonzuso A, Pirri C, et al Non-alcoholic fatty liver disease (NAFLD) and MTHFR 1298A > C gene polymorphism. Eur Rev Med Pharmacol Sci. 2014;18(2):151–159. Epub 2014/02/04. . 24488901

[23] Hu L, Zhang Q, Miao F, Tai J, Liu J. Association of non-alcoholic fatty liver with plasma homocysteine and the methylenetetrahydrofolate reductase gene polymorphism in patients of type 2 diabetes mellitus in Shanxi, China. Chin J Gen Pract. 2009;8(6):385–388.

[24] Chen H, Guo J, Wang R, Hu S, Wu Y, Mao Y. Methylenetetrahydrofolate reductase gene polymorphism in non-alcoholic fatty liver disease. Chinese Journal of General Practice. 2014;12(12).

[25] Serin E, Guclu M, Atac FB, Verdi H, Kayaselcuk F, Ozer B, et al Methylenetetrahydrofolate reductase C677T mutation and nonalcoholic fatty liver disease. Dig Dis Sci. 2007;52(5):1183–1186. Epub 2007/03/16. 10.1007/s10620-006-9565-7 . 17356914

[26] Sazci A, Ergul E, Aygun C, Akpinar G, Senturk O, Hulagu S. Methylenetetrahydrofolate reductase gene polymorphisms in patients with nonalcoholic steatohepatitis (NASH). Cell Biochem Funct. 2008;26(3):291–296. Epub 2007/06/15. 10.1002/cbf.1424 . 17563923

[27] Orlovskiy V, Kuchma N, Murenets N, Orlovskiy A. C677T and A1298C Allele Polymorphism Gene of Methylenetetrahydrafolatereductase in Patients with Nonalcoholic Fatty Liver Disease and Type 2 Diabetes. Georgian Med News. 2015;(247):38–43. Epub 2015/10/21. . 26483372

[28] Kasapoglu B, Turkay C, Yalcin KS, Kosar A, Bozkurt A. MTHFR 677C/T and 1298A/C mutations and non-alcoholic fatty liver disease. Clin Med. 2015;15(3):248–251. Epub 2015/06/03. 10.7861/clinmedicine.15-3-248 . 26031974 PMC4953108

[29] Franco Brochado MJ, Domenici FA, Candolo Martinelli Ade L, Zucoloto S, de Carvalho da Cunha SF, Vannucchi H. Methylenetetrahydrofolate reductase gene polymorphism and serum homocysteine levels in nonalcoholic fatty liver disease. Ann Nutr Metab. 2013;63(3):193–199. Epub 2013/09/21. 10.1159/000353139 . 24051448

[30] de Carvalho SC, Muniz MT, Siqueira MD, Siqueira ER, Gomes AV, Silva KA, et al Plasmatic higher levels of homocysteine in non-alcoholic fatty liver disease (NAFLD). Nutr J. 2013;12:37 Epub 2013/04/04. 10.1186/1475-2891-12-37 ; PubMed Central PMCID: PMCPmc3626579. 23547829 PMC3626579

[31] Lohmueller KE, Pearce CL, Pike M, Lander ES, Hirschhorn JN. Meta-analysis of genetic association studies supports a contribution of common variants to susceptibility to common disease. Nat Genet. 2003;33(2):177–182. Epub 2003/01/14. 10.1038/ng1071 . 12524541

[32] Higgins JP, Thompson SG, Deeks JJ, Altman DG. Measuring inconsistency in meta-analyses. Bmj. 2003;327(7414):557–560. Epub 2003/09/06. 10.1136/bmj.327.7414.557 ; PubMed Central PMCID: PMCPmc192859. 12958120 PMC192859

[33] Thakkinstian A, McElduff P, D'Este C, Duffy D, Attia J. A method for meta-analysis of molecular association studies. Stat Med. 2005;24(9):1291–1306. Epub 2004/11/30. 10.1002/sim.2010 . 15568190

[34] Higgins JP, Thompson SG. Quantifying heterogeneity in a meta-analysis. Stat Med. 2002;21(11):1539–1558. Epub 2002/07/12. 10.1002/sim.1186 . 12111919

[35] Zintzaras E, Ioannidis JP. Heterogeneity testing in meta-analysis of genome searches. Genet Epidemiol. 2005;28(2):123–137. Epub 2004/12/14. 10.1002/gepi.20048 . 15593093

[36] Begg CB, Mazumdar M. Operating characteristics of a rank correlation test for publication bias. Biometrics. 1994;50(4):1088–1101. Epub 1994/12/01. . 7786990

[37] Moher D, Liberati A, Tetzlaff J, Altman DG. Preferred reporting items for systematic reviews and meta-analyses: the PRISMA statement. PLoS Med. 2009;6(7):e1000097 Epub 2009/07/22. 10.1371/journal.pmed.1000097 ; PubMed Central PMCID: PMCPmc2707599. 19621072 PMC2707599

[38] Sun H, Han B, Zhai H, Cheng X, Ma K. Significant association between MTHFR C677T polymorphism and hepatocellular carcinoma risk: a meta-analysis. Tumour Biol. 2014;35(1):189–193. Epub 2013/10/18. 10.1007/s13277-013-1023-5 . 24132589

[39] Sameer AS, Shah ZA, Nissar S, Mudassar S, Siddiqi MA. Risk of colorectal cancer associated with the methylenetetrahydrofolate reductase (MTHFR) C677T polymorphism in the Kashmiri population. Genet Mol Res. 2011;10(2):1200–1210. Epub 2011/07/07. 10.4238/vol10-2gmr1067 . 21732284

[40] Yan L, Zhao L, Long Y, Zou P, Ji G, Gu A, et al Association of the maternal MTHFR C677T polymorphism with susceptibility to neural tube defects in offsprings: evidence from 25 case-control studies. PLoS One. 2012;7(10):e41689 Epub 2012/10/12. 10.1371/journal.pone.0041689 ; PubMed Central PMCID: PMCPmc3463537. 23056169 PMC3463537

[41] Yang L, Hu X, Xu L. Impact of methylenetetrahydrofolate reductase (MTHFR) polymorphisms on methotrexate-induced toxicities in acute lymphoblastic leukemia: a meta-analysis. Tumour Biol. 2012;33(5):1445–1454. Epub 2012/04/25. 10.1007/s13277-012-0395-2 . 22528943

[42] Trovato FM, Catalano D, Ragusa A, Martines GF, Pirri C, Buccheri MA, et al Relationship of MTHFR gene polymorphisms with renal and cardiac disease. World J Nephrol. 2015;4(1):127–137. Epub 2015/02/11. 10.5527/wjn.v4.i1.127 ; PubMed Central PMCID: PMCPmc4317623. 25664255 PMC4317623

[43] Das PM, Singal R. DNA methylation and cancer. J Clin Oncol. 2004;22(22):4632–4642. Epub 2004/11/16. 10.1200/jco.2004.07.151 . 15542813

[44] Ueland PM, Hustad S, Schneede J, Refsum H, Vollset SE. Biological and clinical implications of the MTHFR C677T polymorphism. Trends Pharmacol Sci. 2001;22(4):195–201. Epub 2001/04/03. . 11282420 10.1016/s0165-6147(00)01675-8

[45] Duthie SJ, Narayanan S, Brand GM, Pirie L, Grant G. Impact of folate deficiency on DNA stability. J Nutr. 2002;132(8 Suppl):2444s–2449s. Epub 2002/08/07. . 12163709 10.1093/jn/132.8.2444S

[46] Pereira AC, Schettert IT, Morandini Filho AA, Guerra-Shinohara EM, Krieger JE. Methylenetetrahydrofolate reductase (MTHFR) c677t gene variant modulates the homocysteine folate correlation in a mild folate-deficient population. Clin Chim Acta. 2004;340(1–2):99–105. Epub 2004/01/22. . 14734201 10.1016/j.cccn.2003.09.016

[47] Friso S, Choi SW, Girelli D, Mason JB, Dolnikowski GG, Bagley PJ, et al A common mutation in the 5,10-methylenetetrahydrofolate reductase gene affects genomic DNA methylation through an interaction with folate status. Proc Natl Acad Sci U S A. 2002;99(8):5606–5611. Epub 2002/04/04. 10.1073/pnas.062066299 ; PubMed Central PMCID: PMCPmc122817. 11929966 PMC122817

[48] Liang TJ, Liu H, Zhao XQ, Tan YR, Jing K, Qin CY. Quantitative assessment of the association between MTHFR rs1801131 polymorphism and risk of liver cancer. Tumour Biol. 2014;35(1):339–343. Epub 2013/09/10. 10.1007/s13277-013-1046-y . 24014085

[49] Qi YH, Yao LP, Cui GB, Liang J, Shao QJ, Yan LF, et al Meta-analysis of MTHFR C677T and A1298C gene polymorphisms: association with the risk of hepatocellular carcinoma. Clin Res Hepatol Gastroenterol. 2014;38(2):172–180. Epub 2013/12/10. 10.1016/j.clinre.2013.10.002 . 24316043

[50] Than NN, Newsome PN. A concise review of non-alcoholic fatty liver disease. Atherosclerosis. 2015;239(1):192–202. Epub 2015/01/27. 10.1016/j.atherosclerosis.2015.01.001 . 25617860

[51] Loomba R, Sanyal AJ. The global NAFLD epidemic. Nat Rev Gastroenterol Hepatol. 2013;10(11):686–690. Epub 2013/09/18. 10.1038/nrgastro.2013.171 . 24042449

[52] Neuschwander-Tetri BA, Caldwell SH. Nonalcoholic steatohepatitis: summary of an AASLD Single Topic Conference. Hepatology. 2003;37(5):1202–1219. Epub 2003/04/30. 10.1053/jhep.2003.50193 . 12717402

[53] Basaranoglu M, Kayacetin S, Yilmaz N, Kayacetin E, Tarcin O, Sonsuz A. Understanding mechanisms of the pathogenesis of nonalcoholic fatty liver disease. World J Gastroenterol. 2010;16(18):2223–2226. Epub 2010/05/12. ; PubMed Central PMCID: PMCPmc2868214. 20458758 10.3748/wjg.v16.i18.2223 PMC2868214

[54] Armstrong MJ, Adams LA, Canbay A, Syn WK. Extrahepatic complications of nonalcoholic fatty liver disease. Hepatology. 2014;59(3):1174–1197. Epub 2013/09/05. 10.1002/hep.26717 . 24002776

[55] Zhou TB, Drummen GP, Jiang ZP, Li HY. Methylenetetrahydrofolate reductase (MTHFR) C677T gene polymorphism and diabetic nephropathy susceptibility in patients with type 2 diabetes mellitus. Ren Fail. 2015;37(8):1247–1259. Epub 2015/07/15. 10.3109/0886022x.2015.1064743 . 26161693

[56] Qin X, Shen L, Zhang R, Li Y, Wang X, Wang B, et al Effect of folic acid supplementation on cancer risk among adults with hypertension in China: A randomized clinical trial. Int J Cancer. 2016 Epub 2016/03/19. 10.1002/ijc.30094 . 26991917

[57] Wang Y, Zhang H, Yue S, Zhang K, Wang H, Dong R, et al Evaluation of High Resolution Melting for MTHFR C677T Genotyping in Congenital Heart Disease. PLoS One. 2016;11(3):e0151140 Epub 2016/03/19. 10.1371/journal.pone.0151140 ; PubMed Central PMCID: PMCPmc4798616. 26990189 PMC4798616

