# Supplementary material for: Associations between Methylenetetrahydrofolate Reductase (MTHFR) Polymorphisms and Non-Alcoholic Fatty Liver Disease (NAFLD) Risk: A Meta-Analysis
Source: PLoS One. 2016 Apr 29;11(4):e0154337. doi: 10.1371/journal.pone.0154337 (PMC4851382; doi:10.1371/journal.pone.0154337)
Supplement: S1 Text — (DOCX) [file pone.0154337.s003.docx]

# S1 Text

## Databases searching terms

## (updated to April 1^st^, 2016)

#### Pubmed Database (n=10)

#1：((((((Methylenetetrahydrofolate Reductase (NADPH2)) OR Methylenetetrahydrofolate Reductase (NADPH)) OR Methylene-THF Reductase (NADPH)) OR Methylenetetrahydrofolate Reductase) OR 5,10-Methylenetetrahydrofolate Reductase (NADPH)) OR Methylene Tetrahydrofolate Reductase) OR Tetrahydrofolate Reductase, Methylene -----5036

#2：(((((((((((Non-alcoholic Fatty Liver Disease) OR Non alcoholic Fatty Liver Disease) OR NAFLD) OR Nonalcoholic Fatty Liver Disease) OR Fatty Liver, Nonalcoholic) OR Fatty Livers, Nonalcoholic) OR Nonalcoholic Fatty Liver) OR Nonalcoholic Fatty Livers) OR Nonalcoholic Steatohepatitis) OR Nonalcoholic Steatohepatitides) OR Steatohepatitides, Nonalcoholic) OR Steatohepatitis, Nonalcoholic----- 12742

#1 and #2: (((((((((((((Non-alcoholic Fatty Liver Disease) OR Non alcoholic Fatty Liver Disease) OR NAFLD) OR Nonalcoholic Fatty Liver Disease) OR Fatty Liver, Nonalcoholic) OR Fatty Livers, Nonalcoholic) OR Nonalcoholic Fatty Liver) OR Nonalcoholic Fatty Livers) OR Nonalcoholic Steatohepatitis) OR Nonalcoholic Steatohepatitides) OR Steatohepatitides, Nonalcoholic) OR Steatohepatitis, Nonalcoholic)) AND (((((((Methylenetetrahydrofolate Reductase (NADPH2)) OR Methylenetetrahydrofolate Reductase (NADPH)) OR Methylene-THF Reductase (NADPH)) OR Methylenetetrahydrofolate Reductase) OR 5,10-Methylenetetrahydrofolate Reductase (NADPH)) OR Methylene Tetrahydrofolate Reductase) OR Tetrahydrofolate Reductase, Methylene) -----10

#### EMBASE Database (n=29)

#1 ('Methylenetetrahydrofolate Reductase' OR 'MTHFR') AND ('nonalcoholic fatty liver' OR 'NAFLD' OR 'Nonalcoholic Fatty Liver Disease' OR 'Nonalcoholic Steatohepatitis' OR 'Nonalcoholic Steatohepatitides') AND ('polymorphism' OR 'Genetic Polymorphism' OR 'variants' OR 'variant' OR 'mutation' OR 'mutations' OR 'SNP' OR 'Single Nucleotide Polymorphism') -----32

#1 AND limit 1 to human-----29

#### Cochrane Central Register of Controlled Trials (CENTRAL) Database (n=0)

#1： MeSH descriptor: [Methylenetetrahydrofolate Reductase (NADPH2)] explode all trees-----166

#2： MeSH descriptor: [Polymorphism, Genetic] explode all trees-----3350

#3： MeSH descriptor: [Non-alcoholic Fatty Liver Disease] explode all trees-----134

#4：#1 and #2 and #3-----0

#### Web of Science (WOS) Database (n=24)

TS=((((((((((((((Non-alcoholic Fatty Liver Disease) OR Non alcoholic Fatty Liver Disease) OR NAFLD) OR Nonalcoholic Fatty Liver Disease) OR Fatty Liver, Nonalcoholic) OR Fatty Livers, Nonalcoholic) OR Nonalcoholic Fatty Liver) OR Nonalcoholic Fatty Livers) OR Nonalcoholic Steatohepatitis) OR Nonalcoholic Steatohepatitides) OR Steatohepatitides, Nonalcoholic) OR Steatohepatitis, Nonalcoholic)) AND (((((((Methylenetetrahydrofolate Reductase (NADPH2)) OR Methylenetetrahydrofolate Reductase (NADPH)) OR Methylene-THF Reductase (NADPH)) OR Methylenetetrahydrofolate Reductase) OR 5,10-Methylenetetrahydrofolate Reductase (NADPH)) OR Methylene Tetrahydrofolate Reductase) OR Tetrahydrofolate Reductase, Methylene)) -----24

#### CNKI / WANFANG Database (n=3)

CNKI

主题: MTHFR 基因多态性 非酒精性脂肪肝-----1

WANFANG

mthfr * 基因多态性 * 非酒精性脂肪肝 * Date:-2016-----2

#### Scopus Database (n=144)

('Methylenetetrahydrofolate Reductase' OR 'MTHFR') AND ('nonalcoholic fatty liver disease' OR 'NAFLD') AND 'polymorphisms'-----144

#### EBSCOhost Databases (n=11)

('Methylenetetrahydrofolate Reductase' OR 'MTHFR') AND ('nonalcoholic fatty liver disease' OR 'NAFLD') AND 'polymorphisms'-----11
