# Supplementary material for: Associations between Methylenetetrahydrofolate Reductase (MTHFR) Polymorphisms and Non-Alcoholic Fatty Liver Disease (NAFLD) Risk: A Meta-Analysis
Source: PLoS One. 2016 Apr 29;11(4):e0154337. doi: 10.1371/journal.pone.0154337 (PMC4851382; doi:10.1371/journal.pone.0154337)
Supplement: S2 Text — (DOCX) [file pone.0154337.s004.docx]

# S2 Text

## Full-text articles excluded with reasons

## (n=10)

- **meeting/conference abstracts [1-6]**
- **lack of usable data [7-10]**

1. Wierzbicka A, Socha P, Roszczynko M, Szperl M, Jańczyk W, Niemirska A, et al. Common variant of the ace, but not enos, MTHFR, LDL-R, LPA, CRP, and PPARg gene polymorphism is associated with nonalcoholic fatty liver disease in children. Journal of Pediatric Gastroenterology and Nutrition. 2011;52:E75.

2. Trovato GM, Ragusa A, Catalano D, Martines GF, Tonzuso A, Pirri C, et al. MTHFR 1298 A>C gene polymorphism is associated with Non-Alcoholic Fatty Liver Disease (NAFLD) and Insulin Resistance in well-nourished patients. FASEB Journal. 2012;26.

3. Kruger FC, Fisher LR, Kidd M, Daniels C, Kotze MJ. Correlation between mutations in the HFE gene and alanine transaminase levels may increase the risk of cardiovascular disease events in patients with nash. Gastroenterology. 2012;142(5):S1023.

4. Fisher LR, Pretorius J, Kruger FC, Daniels C, Kidd M, Kotze MJ. Development of a pathology supported genetic testing strategy for improved clinical management of patients with non-alcoholic fatty liver disease (NAFLD). Histopathology. 2012;61:141.

5. Dasarathy J, Varghese R, Kalinina I, Lopez R, McCullough AJ, Dasarathy S. Potential benefit of folate restriction in patients with NAFLD with MTHFR homozygous mutation. Hepatology. 2015;62:1261A-1262A.

6. Chirinos Vega JA, Carreras MP, Fernández-Miranda C, López-Alonso G, Ayala R, Arribas C, et al. Hyperhomocysteinemia and C677T methylenetetrahidrofolate reductase gene polymorphisms in Spanish patients with nonalcoholic fatty liver disease. Journal of Hepatology. 2010;52:S140-S141.

7. Frelut ML, Emery-Fillon N, Guilland JC, Dao HH, De Courcy GP. Alanine amino transferase concentrations are linked to folate intakes and methylenetetrahydrofolate reductase polymorphism in obese adolescent girls. Journal of Pediatric Gastroenterology and Nutrition. 2006;43(2):234-239. doi: 10.1097/01.mpg.0000228110.83616.92.

8. Catalano D, Trovato GM, Ragusa A, Martines GF, Tonzuso A, Pirri C, et al. Non-alcoholic fatty liver disease (NAFLD) and MTHFR 1298A > C gene polymorphism. Eur Rev Med Pharmacol Sci. 2014;18(2):151-159. Epub 2014/02/04. PubMed PMID: 24488901.

9. Assy N, Bekirov I, Mejritsky Y, Solomon L, Szvalb S, Hussein O. Association between thrombotic risk factors and extent of fibrosis in patients with non-alcohol fatty liver diseases. World Journal of Gastroenterology. 2005;11(37):5834-5839.

10. Adinolfi LE, Ingrosso D, Cesaro G, Cimmino A, D'Antò M, Capasso R, et al. Hyperhomocysteinemia and the MTHFR C677T polymorphism promote steatosis and fibrosis in chronic hepatitis C. Hepatology. 2005;41(5):995-1003.
